# Gene coexpression network during ontogeny in the yellow fever mosquito, *Aedes aegypti*

**DOI:** 10.1186/s12864-023-09403-4

**Published:** 2023-06-03

**Authors:** Zhinan Lin, Yuqi Huang, Sihan Liu, Qiwen Huang, Biliang Zhang, Tianpeng Wang, Ziding Zhang, Xiaowei Zhu, Chenghong Liao, Qian Han

**Affiliations:** 1 Laboratory of Tropical Veterinary Medicine and Vector Biology, School of Life Sciences, Hainan University, Haikou, 570228 Hainan China; 2 One Health Institute, Hainan University, Haikou, 570228 Hainan China; 3 Department of Neuroscience, Jockey Club College of Veterinary Medicine and Life Sciences, City University of Hong Kong, Kowloon, 99907 Hong Kong SAR China; 4 State Key Laboratory of Agrobiotechnology, College of Biological Sciences, China Agricultural University, Beijing, 100193 China

**Keywords:** Mosquito, Ontogeny, *Aedes aegypti*, Gene coexpression network, WGCNA

## Abstract

**Background:**

The behaviors and ontogeny of *Aedes aegypti* are closely related to the spread of diseases caused by dengue (DENV), chikungunya (CHIKV), Zika (ZIKV), and yellow fever (YFV) viruses. During the life cycle, *Ae. aegypti* undergoes drastic morphological, metabolic, and functional changes triggered by gene regulation and other molecular mechanisms. Some essential regulatory factors that regulate insect ontogeny have been revealed in other species, but their roles are still poorly investigated in the mosquito.

**Results:**

Our study identified 6 gene modules and their intramodular hub genes that were highly associated with the ontogeny of *Ae. aegypti* in the constructed network. Those modules were found to be enriched in functional roles related to cuticle development, ATP generation, digestion, immunity, pupation control, lectins, and spermatogenesis. Additionally, digestion-related pathways were activated in the larvae and adult females but suppressed in the pupae. The integrated protein‒protein network also identified cilium-related genes. In addition, we verified that the 6 intramodular hub genes encoding proteins such as EcKinase regulating larval molt were only expressed in the larval stage. Quantitative RT‒PCR of the intramodular hub genes gave similar results as the RNA-Seq expression profile, and most hub genes were ontogeny-specifically expressed.

**Conclusions:**

The constructed gene coexpression network provides a useful resource for network-based data mining to identify candidate genes for functional studies. Ultimately, these findings will be key in identifying potential molecular targets for disease control.

**Supplementary Information:**

The online version contains supplementary material available at 10.1186/s12864-023-09403-4.

## Background

Insect ontogeny includes gametogenesis, fertilization, embryogenesis, hatching, metamorphosis, and senescence [1], which represent the insect’s life cycle. The juvenile is for more efficient feeding, and the adult is for more convenient transmission and reproduction. The exoskeleton replacement, molting, and metamorphosis from the juvenile stage to adult stage are mediated or controlled by hormones [1]. A few ontogeny studies have been performed on *Aedes aegypti*. Arylalkalamine N-acetyltransferase (aaNAT) catalyzes the acetylation of arylalkanes such as dopamine to form N-acetylalkylamines with corresponding physiological functions [2]. aaNAT-1 has the highest expression in the *Aedes aegypti* pupal stage, and it has been proven that the enzyme plays an essential role in the pigmentation and metamorphosis of adult mosquitoes in mutation experiments [3]. DOPAL synthase catalyzes L-dopa to produce DOPAL in insects, and the product, DOPAL cross-links with cuticle proteins to form a flexible cuticle with high tensile strength. The DOPAL synthase gene is transcribed first in the soft abdomen of mosquitoes and is essential for morphological changes before and after eclosion [4]. A microarray analysis study of transcripts in the ontogeny of *Ae. aegypti* showed that development-related genes are highly stage-specific in the *Ae. aegypti* life cycle [5].

*Ae. aegypti* is a major vector of arthropod-borne viruses (arboviruses), and YFV, DENV, ZIKV, and CHIKV are the most widespread arboviruses, causing substantial morbidity and mortality among human populations in tropical regions of the world [6]. Due to the lack of treatments, mosquito population control remains the primary way to interrupt the transmission of these viruses [7]. There are chemical control, biological control, and other mosquito control methods, but the subsequent insecticide resistance and species invasion also bring new challenges. The RNA interference (RNAi) method impairs gene expression by degrading RNA into short RNAs that activate ribonucleases to target homologous mRNA [8], and this method has been reported to successfully silence genes of odorant receptors in *Culex quinquefasciatus* [9]. Studying gene expression patterns of *Ae. aegypti* ontogeny may help us identify more gene targets to develop new population control strategies.

At present, new technologies have been applied to study the development of *Ae. aegypti,* e.g., RNA interference and CRISPR gene editing [10, 11]. Compared with DNA microarrays, high-throughput RNA sequencing (RNA-Seq) is a more powerful technique to study gene expression at specific developmental stages [12]. In recent years, RNA-Seq has been widely used to study the biology of *Ae. aegypti*, such as revealing the changes in ovarian gene expression in the female after a blood meal [13] and identifying genes related to sperm storage in the spermatheca of female mosquitoes before and after fertilization, helping to improve the understanding of fertilization and embryogenesis [14]. However, few reports have comprehensively investigated gene expression in the ontogeny of *Ae. aegypti*. Weighted gene coexpression network analysis (WGCNA) is a popular tool to study gene expression patterns with bulk RNA-Seq data and can assign corresponding weights to the correlation between any two genes and then cluster genes with similar expression patterns into the same module. Genes within a module are often considered to have similar biological functions or be part of the same biological pathway [15]. By correlating modules with different developmental stages or traits, key genes for regulating development will be identified [16]. Recently, WGCNA has been used to identify long noncoding RNAs associated with the developmental stages of *Ae. albopictus* [17], and to identify key genes in different wintering stages of *Anoplophora glabripennis* larvae and the Chinese white wax scale insect [18, 19]. While gene function annotation is usually lacking for nonmodel organisms, eggNOG-mapper is a tool developed in recent years to infer functional genome annotation based on the comparison of query proteins with those in functionally known protein databases [20], and has been used for functional genome annotation of some species [21, 22].

In a previous study, Akbari et al. built a developmental transcriptome profile, and their work mostly focused on transposon (TE) and small RNA analysis [23]. With the development of long-read sequencing technology, Matthews et al. published a reannotated AaegL5 genome assembly [24], allowing the mapping results of bulk RNA sequencing reads to significantly increase. Different from the two previous studies, we got the new information about the relations between functional genome and the life stage of *Ae. Aegypti*. We mapped *Ae. aegypti* bulk RNA sequencing data from the above two published papers to the latest reference genome (AaegL 5.0) [24] to construct the ontogeny transcriptional profile. EggNOG-mapper software was used to deduce the functional genome annotation of *Ae. aegypti* by aligning query proteins to known protein databases. WGCNA was used to identify modules and key genes related to ontogeny stages and male gametes. Subsequently, Gene Ontology (GO) and Kyoto Encyclopedia of Genes and Genomes (KEGG) analyses allow us to understand the molecular mechanisms and metabolic pathways involved [25–27]. Differentially expression gene (DEG) analysis [28] was used to compare transcriptome changes between males and females at different pupal stages (early, middle, late pupae), and biological events were identified by gene set enrichment analysis (GSEA) [29]. To verify the result of our analysis, the expression pattern of the intra-modular hub genes of ontogeny-specific modules is examined by quantitative RT PCR. This study provides a basis for the molecular mechanisms involved in the ontogeny of *Aedes aegypti*.

## Results

### Gene coexpression network analysis

**Fig. 1:**
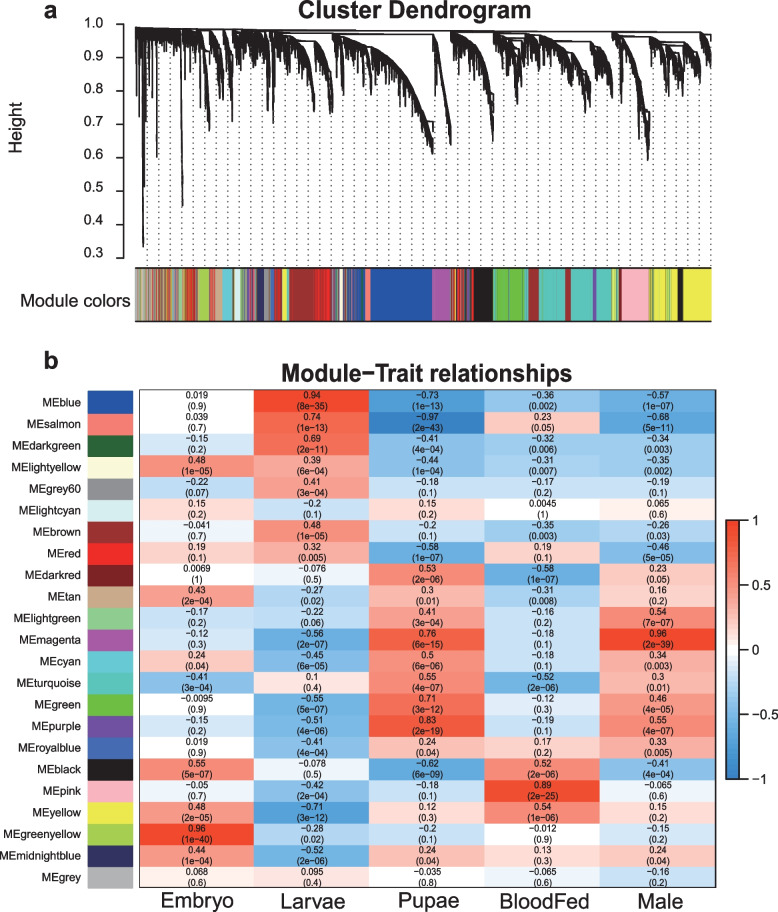
Scale-free weighted gene coexpression network construction analysis. **a** All genes of the network were divided into 23 modules, and each color represents a module; **b** Heatmap showing the correlation between module and ontogeny stages or traits. Each row corresponds to a module. Each column corresponds to an ontogeny stage or trait. A high degree of positively correlation is indicated by dark red (close to 1), and a negatively is dark blue (close to -1)

**Fig. 2:**
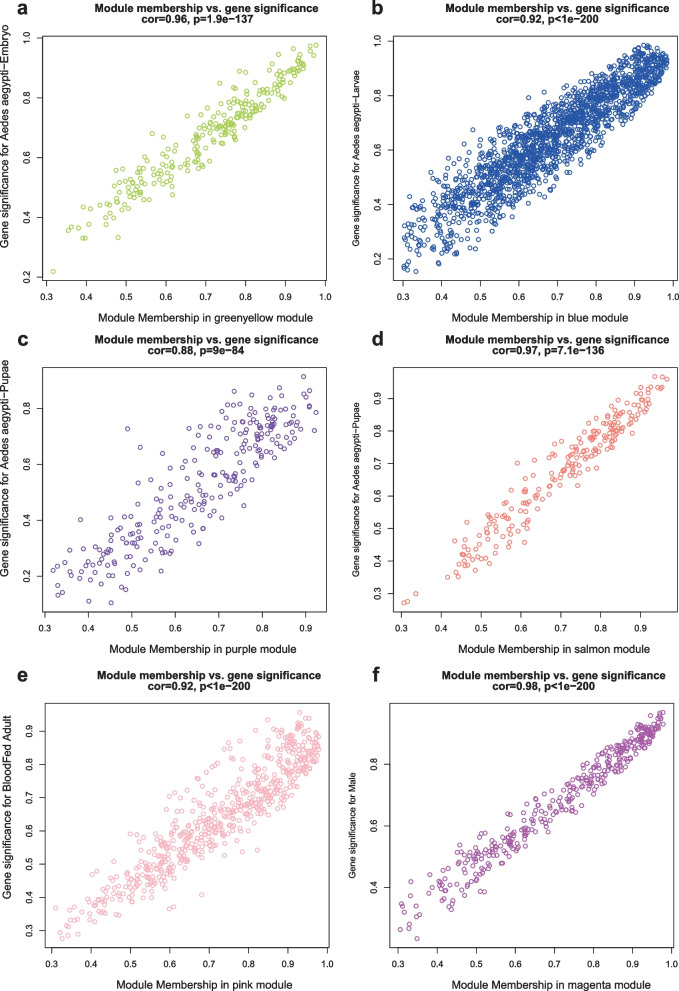
Gene-trait significance (GS) vs. module membership (MM) scatterplots for *Ae. aegypti* ontogeny-associated modules, along with correlation and *P* values indicated. There are highly significant correlations between GS and MM in the six modules (**a**) greenyellow, cor = 0.96, *P* = 1.9e-137; **b** blue, cor = 0.92, *P* < 1e-200; **c** purple, cor = 0.88, *P* = 9e-84; **d** salmon, cor = 0.97, *P* = 7.1e-136; **e** pink, cor = 0.92, *P* < 1e-200; **f** magenta, cor = 0.98, *P* < 1e-200)

Principal component analysis (PCA) of the top 300 variance genes of the normalized TPM expression matrix showed that all samples from different stages could be clustered into their developmental stages, which means that the four distinct stages (embryo, larvae, pupae, and adult samples) were well separated from each other (Additional file 1: Fig. S1). The soft thresholding power (β) value was able to appropriately assess the unsigned scale-free topology of the network according to the WGCNA algorithm. When β is equal to 6, the independence degree of the coexpression network was greater than 0.85 and had a proper mean connectivity (Additional file 1: Fig. S1). In the constructed scale-free weighted gene coexpression network, the remaining 11,664 genes after filtering were divided into 23 modules, with each color representing a module, and the 350 genes in the gray module could not be further divided into any modules (Fig. 1a). The WGCNA identified 6 modules significantly associated with the ontogeny of *Ae. aegypti* (Pearson correlation r > 0.8, *P* < 0.05). For each module, we used red or blue colors to indicate a positive or negative correlation with respect to ontogeny stages, with darker colors indicating a stronger correlation. Amongst them, the greenyellow, blue, purple, pink, and magenta modules are positively correlated with the ontogeny stage, embryos, larvae, pupae, female adults (blood-fed) and males, respectively; and the salmon module was negatively correlated with the pupal stage (Fig. 1b). Subsequently, we calculated the gene significance (GS) and module membership (MM) values of all genes within a module. We found that GS and MM were highly positively correlated (cor = 0.88 ~ 0.98) in these modules and demonstrated that the genes most significantly associated with a trait are often the most critical for the respective module (Fig. 2). Highly connected genes in a module are referred to as intramodular hub genes. These hub genes are considered functionally significant in the enriched functions of the modules. Following the hypothesis that higher connectivity for a gene implies more importance in the module’s functional role, genes with the highest connectivity in the 6 stage specific modules were determined and considered to be the hub genes. Hub genes for the 6 modules and their encoding proteins are described in Table 1.

**Table 1:** Identified hub genes and their encoding proteins for the 6 modules

Module	Hub gene	Description
Greenyellow	*LOC5571533*	Cytochrome P450 6A1
Blue	*LOC5573570*	EcKinase
Purple	*LOC5568197*	Receptor expression-enhancing protein 2
Salmon	*LOC5570211*	Aminopeptidase N
Pink	*LOC5570845*	Kallikrein 1-related peptidase b3
Magenta	*LOC110679324*	Uncharacterized protein

### Functional analysis of six stage-specific modules

In the Gene Ontology analysis, the gene list was enriched into three categories, including biological process (BP), cellular component (CC), and molecular function (MF). We identified 20, 133, 242, 55, 12 and 39 significantly enriched GO terms (BH-adjusted *p*-values < 0.05) in the greenyellow, blue, purple, salmon, pink, and magenta modules, respectively (Additional file 2: Table S1). To find the most enriched events or functions, the GO terms were ranked in ascending order according to their *P*-adjusted value.

The greenyellow module was highly associated with eggs. GO enrichment analysis showed that the significantly enriched terms for the greenyellow module were mainly assigned to BP and MF categories, including ‘chitin-based cuticle development’ in BP, ‘extracellular matrix’ in CC, and ‘structural constituent of chitin-based larval cuticle’ in MF. The results suggested that chitin is a key structural component of cuticle during the early embryo, providing support and protection to the developing mosquito.

The blue module was mainly related to larvae.The most enriched GO terms in BP, CC, and MF categories were ‘lipid catabolic’, ‘proton-transporting V-type ATPase complex’, and ‘peptidase activity’, respectively, with these terms being mainly related to energy metabolism and developmental regulation. It implied that in the larvae of *Aedes aegyptio,* lipid metabolism is important for energy production and metabolism.

The purple and salmon modules were positively and negatively correlated, respectively, with the pupal stage. For the purple module, the significantly enriched terms included ‘ubiquitin-dependent protein catabolic process’ and ‘ubiquitin ligase complex’. For the salmon module, the significantly enriched terms included ‘alpha-amino acid metabolic process’ and ‘carboxylic acid binding’. During the development, many of the pupal tissues and structures of Aedes aegypti are broken down and reorganized into the structures and tissues of the adult mosquito. This requires the targeted degradation of many specific proteins, and the ubiquitin-dependent protein catabolic process plays a key role in this process.

The pink module was mainly associated with blood-fed females. The enrichment results included ‘carbohydrate metabolic process’ for BP and ‘serine hydrolase activity’ for MF. After took a blood meal, female mosquitoes require a large amount of energy to support the development and maturation of their eggs. Carbohydrate metabolism plays a crucial role in meeting this increased energy demand, as it is responsible for the breakdown and conversion of carbohydrates into energy. Serine hydrolases, as mentioned previously, are involved in the breakdown of carbohydrates and other molecules [30].

**Fig. 3:**
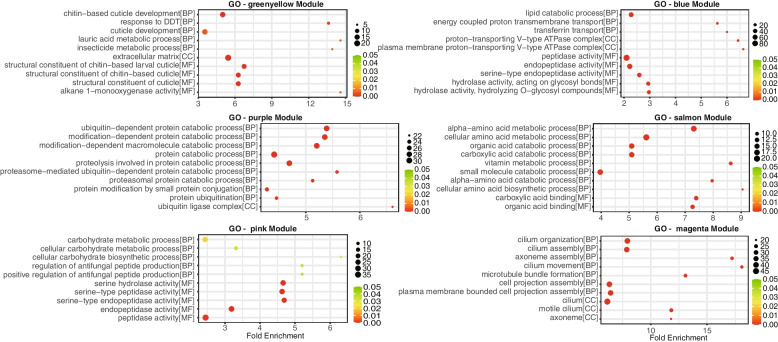
GO enrichment analysis of genes in stage specific modules. The dot symbol represents the number of enriched input genes, and the y-axis means the ratio of the sample frequency representing the number of genes inputted that fall under the same term and background frequency of total genes annotated to that term. Only the 10 most enriched GO terms are shown in this figure (FDR adjusted *P*-adjust < 0.05) and were ranked in ascending order according to *P-adjust* in BP, CC, and MF

In the magenta module correlated to male traits, and the most enriched GO terms in the BP and CC categories were ‘cilium organization’ and ‘cilium’, respectively (Fig. 3). Cilia are hair-like structures found on the surface of many cells, including those of the male reproductive tract. In male *Aedes aegypti*, cilia have been shown to play a crucial role in the transport of sperm from the testes to the seminal vesicles, where they are stored prior to mating [31]. Proper cilium organization is essential for the proper function of these structures.

We performed the KEGG enrichment analysis of genes in the stage-specific modules for further functional categorization. The greenyellow module enriched pathways ‘Primary bile acid biosynthesis’ and ‘PPAR signaling pathway’ have been observed in human studies as regulating early placental development, physiology of clinical pregnancy, and prenatal complications [32, 33]. The primary bile acid biosynthesis pathway and the PPAR signaling pathway in the embryo reflects their importance in regulating lipid metabolism and energy homeostasis during development [34].

For the blue module, the major enriched metabolism pathways included ‘Peptidases and inhibitors’, ‘Protein digestion and absorption’, ‘Pancreatic secretion’, and ‘Collecting duct acid secretion’, which are pathways that may play a role in the immunity and food intake during larval underwater living.The purple and salmon modules showed a high association with pupal stage. The genes were mainly belonged to the ‘Ubiquitin system’ and ‘AMPK signaling pathway’ pathways. The ‘Cytochrome P450’, ‘Tyrosine metabolism’, and ‘Drug metabolism—other enzymes’ pathways were enriched in genes from the salmon module. These enriched KEGG pathways in pupal were mainly metabolism-related, while some of pathways, such as drug metabolism and cytochrome P450, which related pesticide resistance, made this module different from other modules.

In the pink module, ‘Peptidases and inhibitors’, ‘Lectins’, ‘Peptidases and inhibitors’, and ‘Starch and sucrose metabolism’ were the most significantly enriched pathways. To obtain the nutrients to support egg development, the blood-fed females need to digest and metabolize the blood meal efficiently. The upregulation of the peptidases and carbohydrate metabolism suggested that the increased needs for the breakdown of proteins into smaller peptides and amino acids and the complex carbohydrate into simpler sugars that can be used for energy in the females.

The ‘Cytoskeleton proteins’ pathway was the only enriched pathway for the magenta module (Additional file 3: Table S2) in the male mosquito.. The cytoskeleton is particularly important for the development and function of the male reproductive system [35], including the production and release of sperm. The cytoskeleton is also involved in the movement of sperm within the male reproductive tract and the female reproductive tract during mating [36].

### Differentially expression gene analysis between male and female pupae

**Fig. 4:**
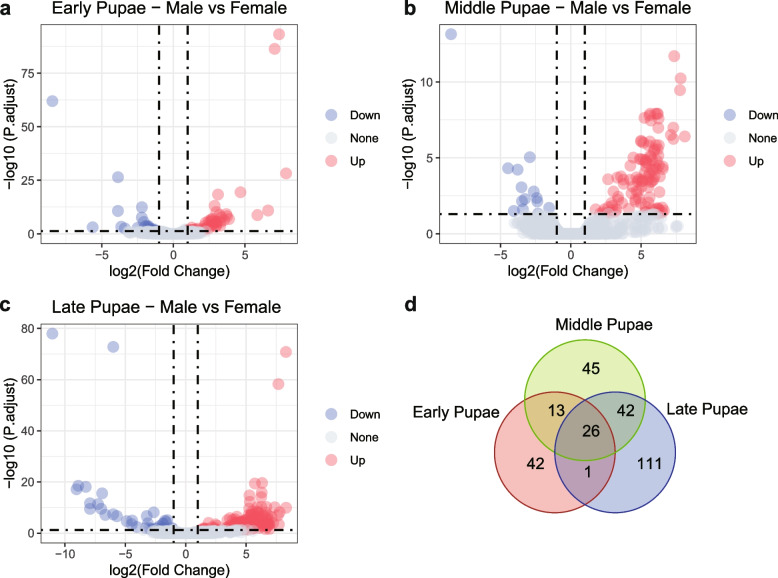
Differential expression analysis between male and female pupae. **a**, **b**, **c** Volcano plot of the differential expression analysis of early, middle, and late pupae, respectively. The red dot means significantly upregulated in males (BH adjusted *P*-value < 0.05 and log_2_FC > 1), and the blue dot indicates significant downregulation in males (BH adjusted *P*-value < 0.05 and log_2_FC < -1). **d** The shared differential expression genes (BH adjusted *P*-value < 0.05 and absolutely log_2_FC > 1) among the three group comparisons

We used DEG analysis for gene expression analysis among male and female pupae to explore the male formation process. The RNA-Seq data from pupae was divided into three groups of comparisons: early, middle, and late male or female pupa. (Fig. 4a, b, c). We identified 82, 126, and 180 DEGs (FDR adjusted *p*-value < 0.05 and absolutely log_2_Foldchange ≥ 1) for early, middle, and late pupae, respectively (Fig. 4d). Following DEG identification, we performed GSEA to translate those DEGs to GO categories, in order to elucidate the functions in which the DEGs participate. The positive normalized enrichment score (NES) of the GO terms in the GSEA results indicated upregulation in male pupae. The negative NES indicated downregulation in males, meaning upregulation in females. In early pupae, ‘mitochondrial ATP synthesis coupled electron transport’ and ‘inner mitochondrial membrane protein complex’ were upregulated in males (Additional file 4: Fig. S2). In middle pupae, ‘cilium assembly’ and ‘sperm flagellum’ are upregulated in the males, and the ‘structural constituent of chitin − based larval cuticle’ was upregulated in females. The only enriched cellular component ‘motile cilium’ was upregulated in the late pupae of males. We observed that sperm development began in early male pupae, and that the formation of the chitin-base cuticle might be critical to the reproductive-related function of females.

### Protein‒Protein interaction (PPI) network analysis of DEGs

**Fig. 5:**
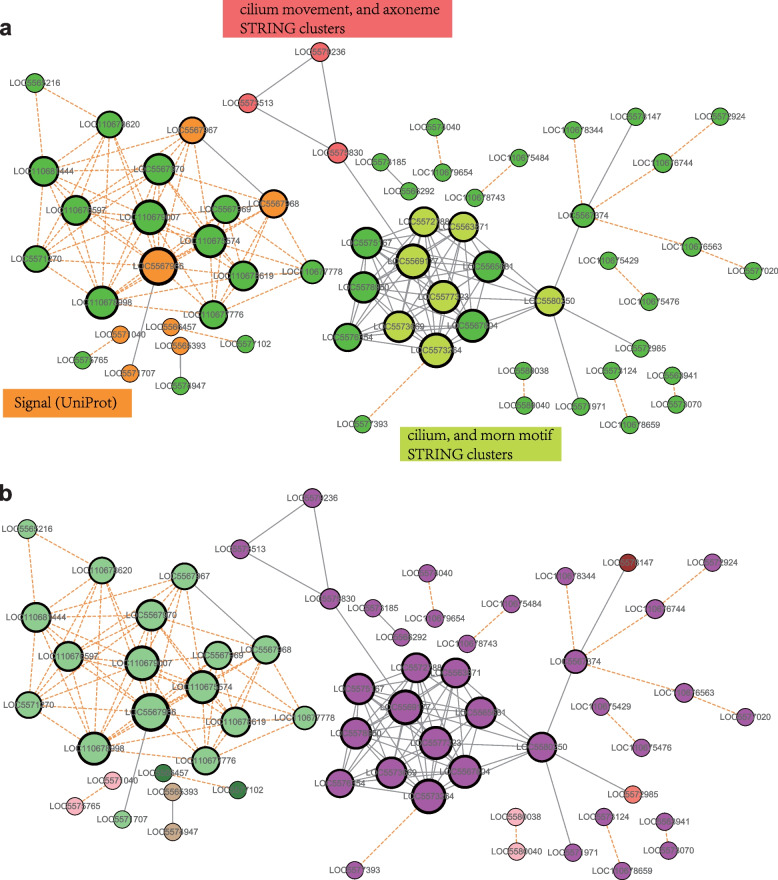
**a** Protein interaction network constructed from all pupae DEG set. Network subclusters with functional annotations are shown in different colors. Key signaling hubs with high betweenness centrality stand out as node size increases. **b** Same as (**A**), but genes are colored according to WGCNA module membership

We noted that magenta coexpression module generally corresponds to the all DEGs from the pupae differential expression analysis. To investigate how these genes interact, we constructed a combined protein interaction network (Fig. 5b). Sixty-two of 280 genes in all DEGs were highly connected within the network. The STRING database enriched clusters including ‘cilium movement, and axoneme’ and ‘cilium, and morn motif’ (Fig. 5a), these cilium-related genes may play an essential role in male sperm development and sex determination. We used the gene symbols beginning with “LOC” here to ensure that the record of those genes, regardless of retroactive or future changes, can still be retrieved on the NCBI web by using "LOC" gene symbols as a query.

### Quantitative RT‒PCR validation

**Fig. 6:**
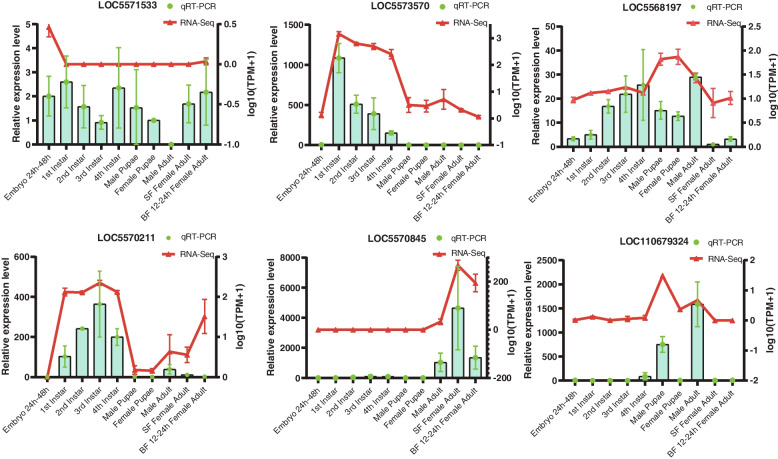
Quantitative PCR (qPCR) validation of intramodular hub genes (**A**-**F**). The qPCR results (green columns) are compared with RNA-Seq data (red lines). The relative expression level of qPCR is shown on the Y axis to the left, and the normalized expression level (TPM) of RNA-Seq is indicated on the Y axis to the right. The ontogeny stage in each graph with the lowest expression level was set as the control group

Quantitative RT‒PCR analysis was performed to validate the RNA-seq data. Six intramodular hub genes were analyzed, including the *Cytochrome P450 6A1* gene (*LOC5571533*), *EcKinase* gene (*LOC5573570*), *receptor expression-enhancing protein 2* gene (LOC5568197), *aminopeptidase N* gene (*LOC5570211*), *Kallikrein 1-related peptidase b3* gene (*LOC5570845*), and *LOC110679324*. The results showed that the *EcKinase* gene was mainly expressed in the larval stages, and that its expression level decreases with the progression of instars. The *aminopeptidase N* gene is expressed in both larva and adult, but its expression level at the pupal stage was very low or even not detectable. The expression level of the *Kallikrein 1-related peptidase b3* gene was expressed in adults and reached to peak in sugar-fed female. However, *LOC110679324* was only expressed in male pupae and male adults, and probably was related to male characteristic development. In general, the expression patterns were in accord with those obtained by RNA-Seq (Fig. 6).

## Discussion

*Ae. aegypti* is considered a major vector of mosquito-borne diseases [37], and these diseases cause hundreds of thousands of deaths per year [38]. The complete metamorphosis of *Ae. aegypti* entails four distinct life stages: egg, larva, pupa, and adult [39]. Notably, previous studies have not focused on the relations between the functional genome and the life stage of *Ae. aegypti* [23, 24]. Thus, understanding the molecular mechanisms during ontogeny may help us develop efficient strategies for controlling the mosquito populations. In this study, transcriptomes covering the whole *Ae. aegypti* ontogeny process and WGCNA were used to construct the weighted gene coexpression network. The resulting coexpression network analysis allowed the identification of stage-specific modules (gene clusters) during ontogeny, as did the enrichment analysis in the GO and KEGG annotation. Highly connected genes in a module (intramodular hub genes) were also determined, as they are key drivers of a molecular process or act as a representative of the predominant biological function of the module [16, 40]. By construct an integration network of coexpression and protein‒protein interaction, we were able to gain a clearer picture of the intrinsic nature of male gametogenesis and sex determination.

The late embryo stage was significantly positively correlated with the greenyellow module. Sixteen genes in this module were enriched to the ‘chitin-based cuticle development’ biological process, including *LOC5570782* (*Chitotriosidase-1*) and *LOC5564327* (*Transcription factor SOX-3*) and cuticle genes. In this biological process, *chitotriosidase-1* is a chitinase that degrades chitin, and the *transcription factor SOX-3* is known to regulate embryonic brain development in humans [41]. The embryonic cuticle of most insects is a chitin-based structure, and chitin deposition occurs later in embryogenesis, which helps the first instar larva hatch from the chorion [42]. There is some evidence showing that interfering with the chitin synthase and chitinase genes will block the development from pro-larva to first instar larva [43, 44]. In addition, the intro-modular hub gene *LOC5571533* (*Cytochrome P450 6A1*) was assigned to the ‘response to DDT’ and ‘alkane 1-monooxygenase activity’ biological process. *Cytochrome P450* genes are related to the morphogenetic processes of the late embryo and regulate the construction of the cuticle layer [45]. Notably, these genes are overexpressed following insecticide exposure in the embryonic stage [46].

After hatching from the egg, mosquito larvae undergo 4 molts before entering the pupal stage, and the larvae grow larger after each molt. During this period, larvae constantly eat since breathing through the siphon tube is a highly energy-consuming process [47]. Gene ontology analysis shows that 56 genes enriched in the ‘lipid catabolic’ process are expressed in whole larva, and that 15 genes enriched to the ‘proton-transporting V-type ATPase complex’ cellular component used the generated ATP to make enough energy for growth. In addition, GO analysis showed 96 genes enriched in the ‘peptidase activity’ molecular function, KEGG analysis enriched the ‘Peptidases and inhibitors’, ‘Protein digestion and absorption’, ‘Pancreatic secretion’, and some immune-related pathways (e.g., ‘Rheumatoid arthritis’, ‘Collecting duct acid secretion’, ‘Vibrio cholerae infection’, and ‘Lysosome’) were enriched. Most of the pathways were related to digestion and the immune system, demonstrating that immune mechanisms and heavy feeding are extremely important for larval survival. Mosquito serine proteases and serine protease homologs have been investigated for a possible role in the digestion process and antiparasitic response [48]. The intramodular hub gene of the blue module *LOC5573570* has been deduced as ecdysteroid 22-kinase (EcKinase) in the Pfam protein database. It is only abundantly expressed in 1–4 instar larvae and has been shown that catalyze the conversion of free ecdysteroids to physiologically inactive ecdysteroid 22-phosphates [49], which may regulate the molts process of mosquito larvae.

When a mosquito larva molts for the fourth time, it becomes a pupa. They float near the surface, diving or swimming in tumbling motions under changing light conditions [50]. The pupae do not eat or molt, and they use trumpets (two siphons) for breathing air, and change into adult mosquito forms inside the casing [51]. For the purple module genes, which were positively related to the pupa stage, the GO and KEGG analyses showed that 29 genes were significantly enriched in ‘ubiquitin-dependent protein catabolic process’ and ‘ubiquitin system’. Ubiquitin is a small regulatory protein that has already been shown to regulate the pupation timing of flies by controlling transcriptional repressor degradation [52]. The annotation of the hub gene *LOC5568197* showed Receptor expression-enhancing protein (REEP) 2, and it was assigned to the ‘presynapse’ cellular component. REEP can enhance the cell surface expression of G protein-coupled receptors (GPCRs) [53], which may be relevant to sensors in adult airborne living. Conversely, the salmon module genes were negatively related to the pupal stage. The GO analysis enriched 18 genes to ‘alpha-amino acid metabolic process’, and the KEGG analysis showed that ‘Cytochrome P450’, ‘Tyrosine metabolism’, and the hub gene *LOC5570211* encoding the aminopeptidase N were enriched. These enriched categories are negatively related to pupae, which indicated they were being suppressed at the pupal stage. By suppressing the metabolism of amino acids such as tyrosine, we ensures that there was not too much degradation of tyrosine. In the previous study, we learned that tyrosine is converted to tanning precursors during larval-pupal metamorphosis [54], and Tyrosine hydroxylase regulates the melanin pathways, and that tyrosine in pupae may play an essential role in mosquito metamorphosis [55]. The integrated PPI network provides the new potential gene interactions for future experiment studies. Meanwhile, the GSEA enrichment results of DEGs in middle pupae were suggested that ‘structural constituent of chitin − based larval cuticle’ was downregulated in males and upregulated in females (Additional file 4: Fig. S2). Female adults have a soft and flexible cuticle to allow movement and blood feeding [10]. While the GSEA results for males mostly identified ‘inner mitochondrial membrane protein complex’ and ‘cilium assembly’ as being enriched in the DEGs of the early and middle pupae, the ‘motile cilium’ was only enriched in the late pupae. The GSEA enrichment results may reveal the sperm formation in the mosquito, Sperm cell deformation occurred in the early pupae, while flagellum assembly mainly occurred in the middle pupae and finally matured in the late pupae.

Once the metamorphosis is complete, a pupa will go to the surface on the water, the back of the casing will split, and an adult mosquito will emerge [56]. Notably, females feed on blood to obtain all nutrients for raising eggs after mating, and humans are one of their blood hosts [57]. In the pink module, the KEGG enriched lectins pathways including *LOC5563672* (*C-type lectin*), *LOC5566555* (cation-independent mannose-6- phosphate receptor), and some fibrinogen and fibronectin genes. C-type lectins have been identified and regarded as a critical component of hematophagous mosquito saliva in innate immunity and in promoting disease transmission in mammals [58], which had a predicted ligand binding specificity for mannose. The GO analysis identified 28 genes as being enriched in the ‘serine hydrolase activity’ molecular function, and the hub gene *LOC5570845* was *kallikrein 1-related peptidase b3*. The kallikrein-kinin system is a poorly understood hormonal system, and we only know that it can regulate bradykinin release in a host animal during blood feeding [59, 60]. Bradykinin and kallidin are vasodilators that act on many cell types and play a role in inflammation, blood pressure control, coagulation, and pain in human research [61]. In the KEGG analysis, the enriched ‘Protein digestion and absorption’ pathway was activated at this stage, and the GO enriched ‘carbohydrate metabolic process’ biological process indicated that the food source are transform to the blood and sugary juice. The sex of mosquitoes can be observed from the pupal stage [62], and we identified the magenta module genes that were significantly associated with male pupae and adulthood. The KEGG and GO analyses showed that ‘cytoskeleton proteins’ and ‘cilium’ were enriched, respectively. Motile cilia are found in the reproductive systems to maintain the suspension of sperm within the intraluminal fluid [63], and PPI network analysis identified a cluster of cilium-related genes may participate in sperm cilium formation. A previous study showed that the leucyl aminopeptidase family encodes the principal protein constituent of *Drosophila* sperm and plays a vital role in spermatogenesis [64]. The intramodular hub gene *LOC110679324* has not been characterized, but its expression profile shows that only abundantly transcription during male pupal to adult stages, and we hope in the future to further explore its function in male pupation and reproductive system in the future.

Currently, chemical insecticides are still the primary approach to control mosquito population, but their misuse has increased insecticide resistance levels among mosquito populations [65]. With the rapid development of biopesticide technologies in recent decades, RNAi has been used as a new pest control tool for mosquito populations. Fei et al. designed an RNAi system by using transgenic microalgae as vectors and fed it to mosquito larvae [66]. Notably, population control for mosquitoes is the principal method to prevent outbreaks of mosquito-borne diseases [67]. Diverse and innovative mosquito control technologies have become increasingly important, and this study provides new insights for biopesticide developers.

## Conclusion

Knowledge of *Ae. aegypti* ontogeny is crucial in identifying key targets to block the transmission of mosquito-borne viruses. In this study, an *Ae. aegypti* coexpression network was constructed, and candidate genes and candidate genes were selected to verified. The results here reveal the correlations between enriched functional terms, the identified intramodular hub genes, and known mosquito biology. Building on the original publications from which the bulk RNA sequencing data were obtained, our discoveries provide new insights into the functional genome of *Ae. aegypti* in different life stages. The identification of the genes may be useful for further understanding the molecular mechanism of critical biological processes during ontogeny *Ae. aegypti* ontogeny. Moreover, the hub genes that encode proteins whose functions are still unknown could be priorities when performing in vitro study.

## Methods

### Data preparation and prep-process of RNA-Sequencing data

The transcriptome data of the *Ae. aegypti* ontogeny stages were downloaded from the Sequence Read Archive (SRA) database of National Center for Biotechnology Information (NCBI) [23, 24]. The SRA accessions and sample information are provided in Additional file 5: Table S3. We downloaded a total of 73 publicly available SRA format files of bulk RNA-Seq. Samples are from the late embryo, the 1^st^-4^th^ instar larvae, pupae, and male, female and blood-fed adults. The fastq format files of RNA-Seq were extracted from SRA files using Fastq-dump software (v2.11.0) [68], and Fastp software (v0.23.1) was used to perform quality control of reads [69]. The trimming process removed read adapters, and sequences shorter than 15 bases were removed to ensure that only 40% of the bases in each read had a quality value lower than 15.

### Reads quantification

HISAT2 [70] software (v2.1.0) was used for mapping reads (both paired-end and single-end) to the genome (AaegL5.0, NCBI genome), and featureCounts [71] software (v2.0.1) was used to obtain a count matrix by counting reads of each gene. This study only focused on protein-coding genes and their functional analysis. The count matrix was converted to the Transcripts Per Million (TPM) matrix to eliminate the effect of gene length and batch effect on the number of read. we provide the transcriptome profile TPM matrix of *Ae. aegypti* ontogeny in Additional file 6: Dataset S1.

### Functional genome annotation

The *Ae. aegypti* functional genome (protein-coding gene) was downloaded from NCBI and eggNOG-mapper [72] software (v2) was run to deduce its annotation. This software uses the HMMER algorithm to align the query sequence to the hidden Markov model (HMM) database and obtain the matching OGs (orthologous groups) results, which are then aligned with all protein sequences in the optimal OG to obtain the optimal matching sequence (seed ortholog) result. Briefly, we obtained a functional genome annotation with GO (Gene Ontology) and KEGG (Kyoto Encyclopedia of Genes and Genomes) annotations, which is provided in Additional file 7: Dataset S2.

### Construction of gene co-expression network

The TPM matrix was used to quantify gene expression levels in R v4.1.0. To reduce background noise, the genes in all samples with low expression levels (expression < 1 and coefficient of variation < 0.1) were removed using the filterByExpr function of the edgeR [73] package (v3.34.1). Sample quality was assessed using Principal Component Analysis (PCA) in pcaExplorer [74] package (v2.24.0). The TPM matrix was log_2_ transformed to fit the normal distribution for downstream analysis. The WGCNA [16] (v1.71) package was used to build the unsigned scale-free networks and co-expression modules to identify genes with similar expression patterns. After calculating the Pearson correlation for each pair of genes, the genes were clustered by a topological overlap measure (TOM) matrix [75]. After filtering the low-expression genes, 11,664 genes were included in the weighted gene co-expression network construction and clustered into different modules. In the WGCNA package, the pickSoftThreshold function was used to pick an appropriate soft thresholding power $\beta$ with R^^2^ over 0.8 and mean connectivity over 100. Then the blockwiseModules function was used to construct a coexpression network with a minimum module size of 30. Hub genes with the highest connectivity in each module were identified using the chooseTopHubInEachModule function.

### Functional enrichment analysis

The ontogeny stage-specific modules were identified based on the module-trait relationship (correlation of each module eigengene and traits) and the correlation between gene significance (GS) and module membership (MM) values. The stage-specific modules should have significantly correlated GS and MM values (*P* < 0.05) and high module-trait relationships (correlation coefficient > 0.8). We also used clusterProfiler package (v4.0.5) [76] to perform Gene Ontology (GO) enrichment analysis, including Molecular Function (MF), Cellular Component (CC), and Biological Process (BP), and KEGG pathway enrichment analysis.

### Differential expression gene analysis

The limma package [28] was used for differential expression analysis between males and females in the early, middle, and late pupae stages. We draw the volcano plots to show the DEG results with the ggplot2 package (v3.4.0) [77], the genes with absolutely log_2_FoldChange ≥ 1 and adjusted *p*-values (FDR) < 0.05 were identified to be significantly differentially expressed. To further understand the biological events in gender development, the DEG analysis results were trait as input for gene set enrichment analysis (GSEA) with clusterProfiler package.

### Construction of protein‒protein interaction (PPI) network

The protein‒protein interaction (PPI) network of significantly differentially expressed genes (DEGs) was identified in the previous step and constructed by using the Search Tool for the Retrieval of Interacting Genes (STRING) database (http://string-db.org/). All significant DEGs of the early, middle, and late pupae stages were combined, and then the STRING database was searched to obtain the PPI network from known interactions. The interaction with medium confidence (combined score > 0.4) was deemed to be statistically significant. The remaining unconnected genes from the DEG set were then added to the network based on their correlation with any of the genes already present in the network requiring Pearson r ≥ 0.96. The resulting interaction network was then visualized in Cytoscape (v3.5.1), and the WGCNA module colors were then integrated into the PPI network.

### Quantitative RT-PCR analysis

To validate the gene expression patterns of RNA-Seq, the expression levels of 6 intra-modular genes were analyzed by quantitative RT-PCR (qPCR). Mosquitoes of ten developmental stages were collected to extract total RNA by using Total RNA Extractor (Sangon, Shanghai, China) and reverse transcribed with SPARKscipt II RT Plus kit (With gDNA Eraser) (Shandong Sparkjade Biotechnology Co., Ltd.). Ae-rps17 was used as the internal control to normalize the gene expression level. Six candidate genes primers (Additional file 8: Table S4) were designed using the online website Primer Quest (http://www.idtdna.com/Primerquest/) and primers specificity analysis was performed using online tool Primer-BLAST (http://www.ncbi.nlm.nih.gov/tools/primer-blast/). The qPCR was performed on a LightCycler 96 (Roche) instrument with SYBR Green qPCR Mix (Shandong Sparkjade Biotechnology Co., Ltd.). Each qPCR volume was 10 μL containing 5 μL of SYBR Green qPCR Mix, 0.5 μL of each primer, 1 μL of cDNA template, and 3 μL of ddH_2_O. Data from three biological replicates and three technical replicates for ten ontogeny stages from LC96 software were analyzed for the differences.

## Supplementary Information

**Additional file 1: Figure S1. **Preprocessing of the ontogeny expression profile before construction of the coexpression network. (a) Principal component analysis of the RNA-Seq sample; (b) The scale independence and mean connectivity of co-expression network in different soft threshold (β), and the red line represent the R^2=0.8.

**Additional file 2: Table S1. **GO enrichment results of the ontogeny-specific module genes.

**Additional file 3: Table S2. **KEGG enrichment results of the ontogeny-specific module genes.

**Additional file 4: Figure S2.** (a) GSEA results of GO categories for early pupae differential expression analysis between males and females; (b) GSEA results of GO categories for middle pupae differential expression analysis between males and females; (c) GSEA results of GO categories for late pupae differential expression analysis between males and females. A positive normalized enrichment score (NES) indicates upregulation in male pupae, and a negative NES indicates downregulation in males, indicating upregulation in females.

**Additional file 5: Table S3. **SRA accession of the RNA-Seq sample.

**Additional file 6: Dataset S1.** TPM expression matrix of all ontogeny stage RNA-Seq sample.

**Additional file 7: Dataset S2. **The functional genome annotation was annotated by eggnog-mapper software.

**Additional file 8: Table S4. **qPCR quantification experiment primers of the intra-modular hub genes.

## Data Availability

All data are available in the main text or the supplementary materials.
