# Supplementary figures and images for: Gene coexpression network during ontogeny in the yellow fever mosquito, Aedes aegypti
Source: BMC Genomics. 2023 Jun 3;24:301. doi: 10.1186/s12864-023-09403-4 (PMC10239134; doi:10.1186/s12864-023-09403-4)

a

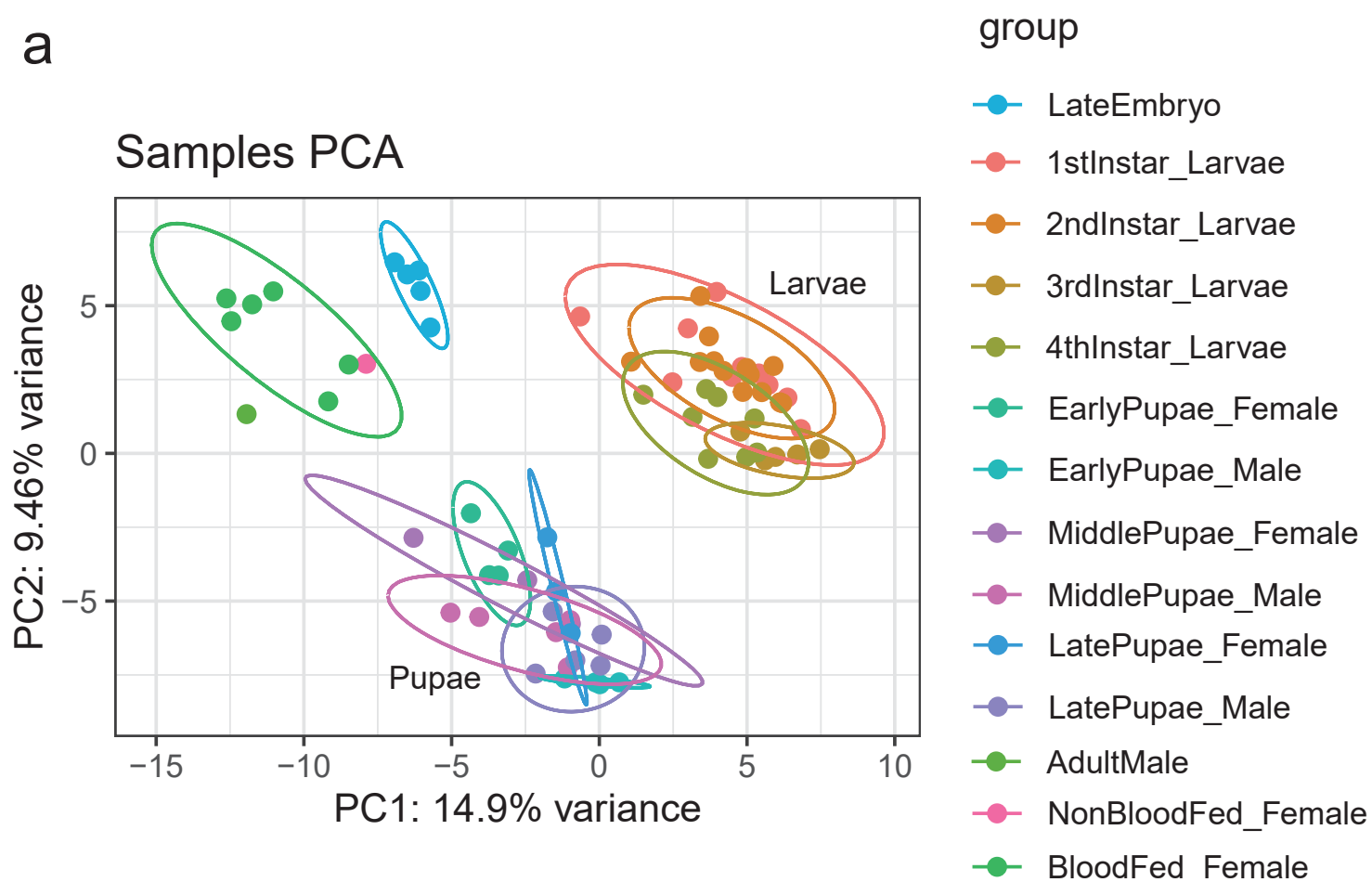

b

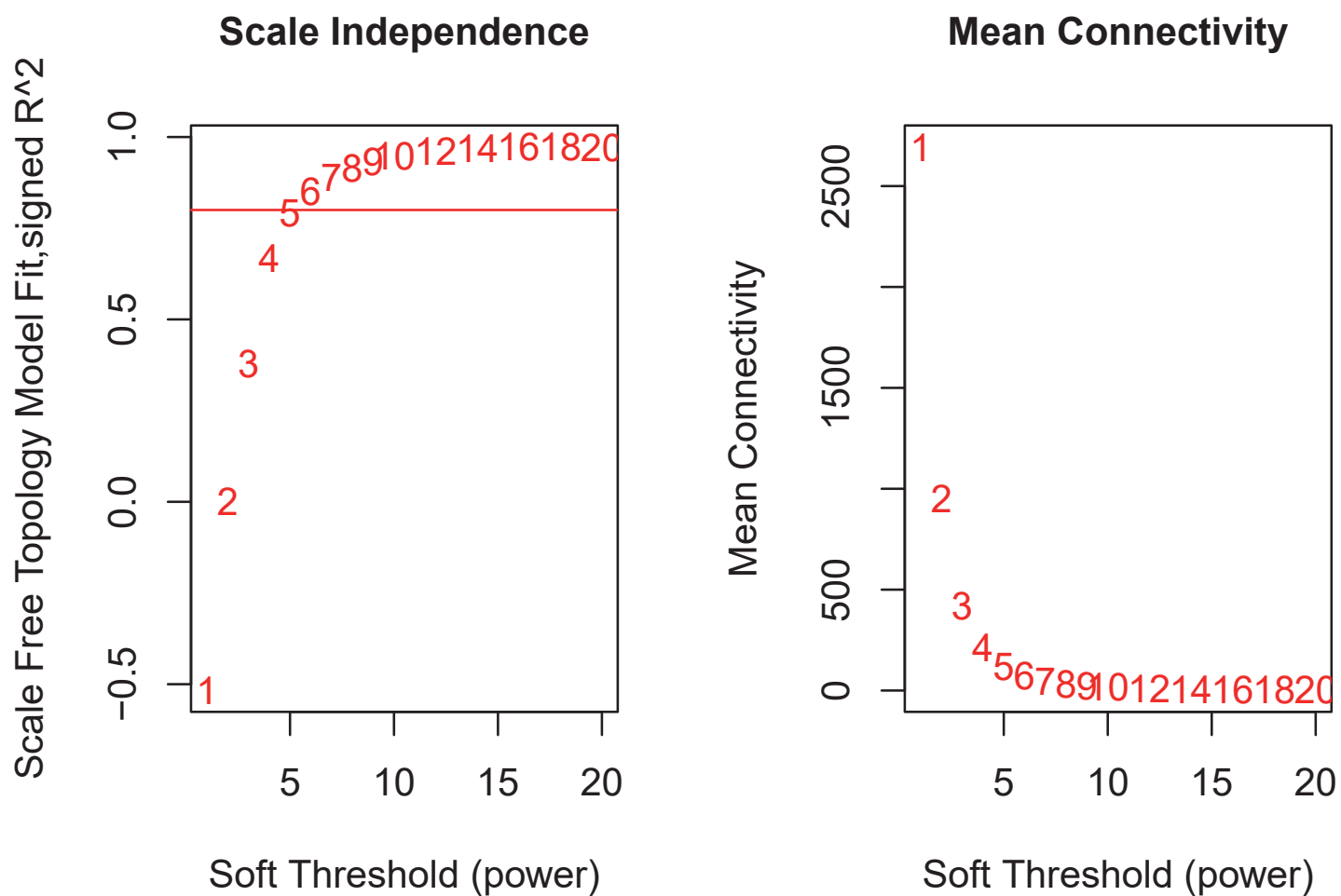

Supplement: Supplementary file 1 — Additional file 1: Figure S1. Preprocessing of the ontogeny expression profile before construction of the coexpression network. (a) Principal component analysis of the RNA-Seq sample; (b) The scale independence and mean connectivity of co-expression network in different soft threshold (β), and the red line represent the R^2=0.8. [file 12864_2023_9403_MOESM1_ESM.pdf]

**a**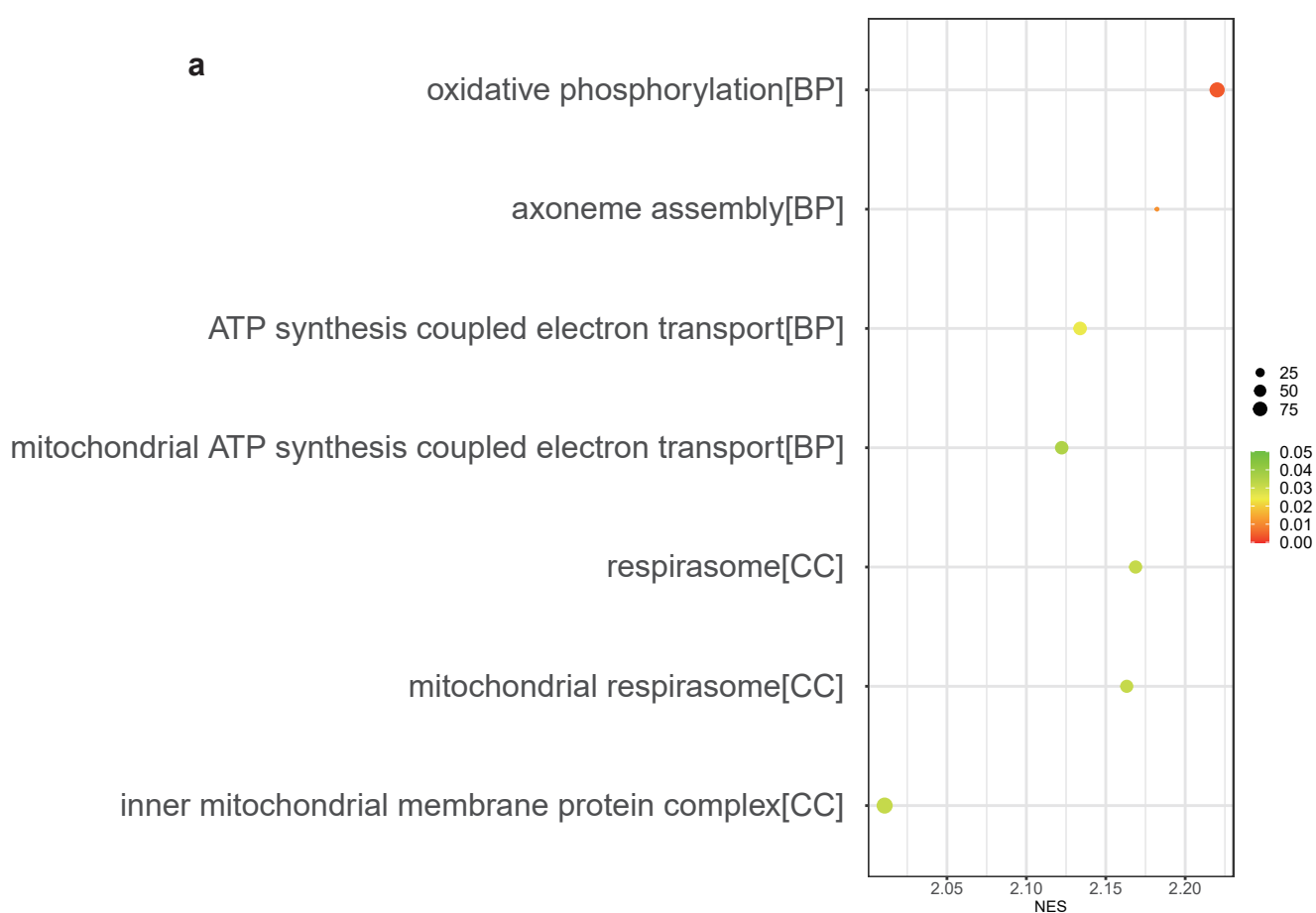**b**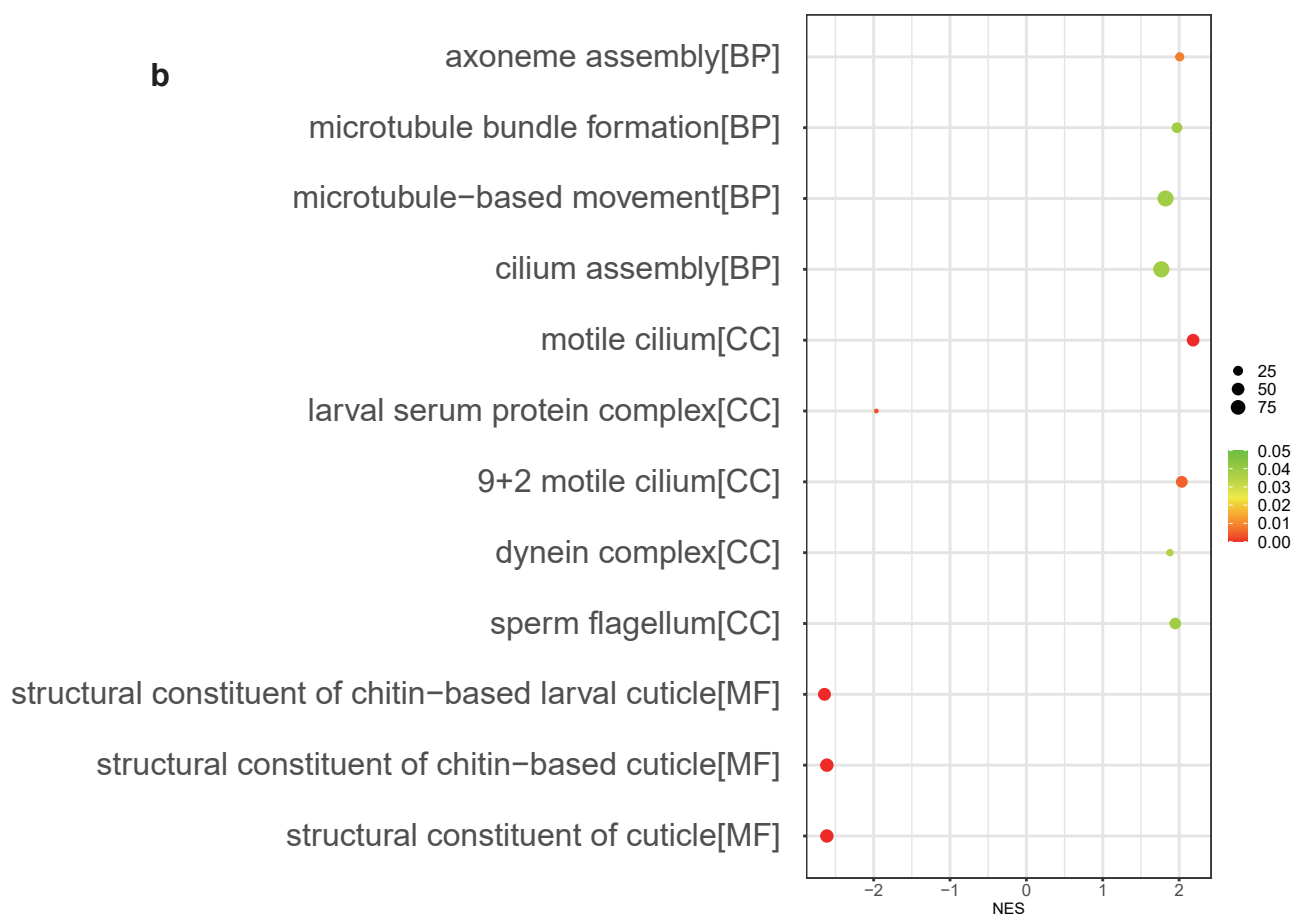**c**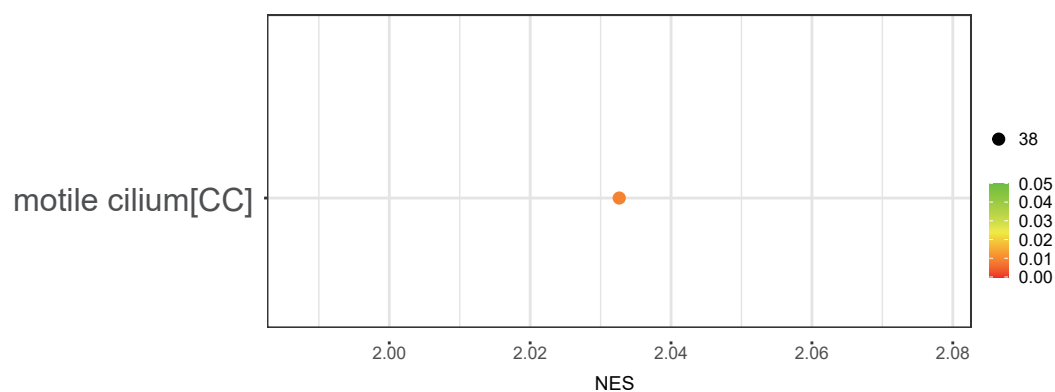

Supplement: Supplementary file 4 — Additional file 4: Figure S2. (a) GSEA results of GO categories for early pupae differential expression analysis between males and females; (b) GSEA results of GO categories for middle pupae differential expression analysis between males and females; (c) GSEA results of GO categories for late pupae differential expression analysis between males and females. A positive normalized enrichment score (NES) indicates upregulation in male pupae, and a negative NES indicates downregulation in males, indicating upregulation in females. [file 12864_2023_9403_MOESM4_ESM.pdf]
